# Assessing the Role of Topical Vancomycin in Reducing Surgical Site Infections Following Spinal Surgery: A Comprehensive Review

**DOI:** 10.7759/cureus.83307

**Published:** 2025-05-01

**Authors:** Rohan Gupta, Matthew Holden

**Affiliations:** 1 Neurosurgery, The University of Edinburgh, Edinburgh, GBR; 2 Public Health, University of St Andrews, St Andrews, GBR; 3 Epidemiology and Public Health, University of St Andrews, St Andrews, GBR

**Keywords:** spine surgery, surgical site infections (ssi), topical antibiotics, topical vancomycin, vancomycin powder

## Abstract

Surgical site infections (SSIs) are a potential complication that can occur after spinal surgery and significantly impact patients’ well-being and healthcare systems. Topical vancomycin has become an attractive low-cost intervention to prevent SSIs in spinal surgery. However, in current literature, the efficacy of topical vancomycin in preventing SSIs is a well-debated topic with conflicting views. The primary objective of this review was to analyze relevant studies to evaluate whether vancomycin powder is an effective intervention in preventing SSIs following spinal surgery.

A widespread electronic literature search was performed using three primary databases: Medline, Embase, and Web of Science. Relevant studies were screened using pre-defined inclusion and exclusion criteria focusing on outcomes of postoperative SSI incidence rates. Eligible articles should have set control groups of patients receiving only standard systemic antibiotics and treatment groups receiving topical vancomycin plus systemic prophylaxis. Data were extracted from included studies, and quality assessment was done, including the risk of bias.

After full-text evaluation, 12 studies were included in this review, with a total of 9,224 patients. Three were randomized controlled trials (RCTs), two were prospective cohort, and seven were retrospective cohort studies. A total of 279 patients developed SSIs, with 194 SSIs in the control groups. Five studies demonstrated a statistically significant decrease in SSI rates with the application of vancomycin powder. Three studies showed a statistically insignificant reduction in SSI rates between vancomycin and control groups, but one presented a statistically significant decrease after propensity score matching of patients. The final four studies showed no statistically significant difference in SSI rates between both study arms. Therefore, seven studies supported the beneficial use of vancomycin powder in preventing SSIs following spinal surgery, while the other five studies had opposing views. Risk of bias was common throughout the studies, including confounding factors, selection and performance biases, and bias due to incomplete data.

Overall, these findings signify the necessity of further well-designed RCTs with double blinding to confirm the effectiveness of topical vancomycin in preventing SSIs during spinal surgery. Clinicians may consider the application of vancomycin powder with caution and on a case-to-case basis.

## Introduction and background

Surgical site infections (SSIs) following spinal surgery are significant complications that burden patients, their families, and the healthcare system. Despite prophylactic systemic antibiotics and intraoperative aseptic techniques, SSIs remain a major complication post-spinal surgery. 

Definition of surgical site infection

SSIs are healthcare-associated infections at the incision site after surgery, accounting for up to 16% of all healthcare-associated infections (HAIs) [1]. The Centers for Disease Control and Prevention (CDC) define SSIs as infections that occur within 30 days post-procedure or 90 days if an implant is used [2,3]. Clinical signs include fever, erythema at the site, drainage of cloudy fluid, and elevated inflammatory markers [4]. The incidence of SSIs following spinal surgery ranges from 0.7% to 12%, making it the third most common complication after pneumonia and urinary tract infections [5,6]. This wide variation is influenced by factors such as surgical method, patient characteristics (age, comorbidities, instrumentation use), operation length, and postoperative cerebrospinal fluid (CSF) leaks [7]. Socioeconomic status also affects infection rates, with lower-income countries reporting significantly higher prevalence of HAIs, including SSIs [8,9].

Aetiology of surgical site infections

SSI risk factors can be categorized into patient, procedural, and microbial factors. Common individual risk factors include diabetes mellitus, obesity, tobacco use, alcohol abuse, malnutrition, old age, cancer, and excessive blood loss [10,11]. Procedural factors encompass instrumentation, surgery duration, poor surgical technique, and history of previous SSIs [10,11]. Untreated SSIs can lead to severe complications, including neurological dysfunction, spinal instability, sepsis, or death [12,13]. The predominant organisms responsible for SSIs after spinal surgery are Gram-positive bacteria, with *Staphylococcus aureus* causing 45.2% and *Staphylococcus epidermidis* causing 31.4% of infections [14]. Methicillin-resistant pathogens are significant, accounting for 34.3% of infections, particularly in revision surgeries [14]. Patients with SSIs caused by methicillin-resistant *Staphylococcus aureus* (MRSA) have higher rates of hospital readmission and increased mortality [15].

Types of spine surgery and infection risk

Spine surgery, often a last-resort treatment after non-surgical options, includes spinal laminectomy, discectomy, and spinal fusion [16]. Infection rates vary by procedure: posterior laminectomy or discectomy carries about a 1% infection risk, while spinal fusion ranges from 2% to 5% [17]. Adding instrumentation to spinal fusion increases the infection risk from 2.4% to 8.5% [17]. The proximity to the spinal cord and surrounding neural structures, as well as the use of hardware, increases the likelihood of SSIs in these procedures. In an effort to reduce infection rates, topical vancomycin has been proposed as a potential adjunct to conventional infection prevention strategies.

Current prevention strategies

Preventing SSIs in spinal surgery is critical for reducing healthcare costs. The National Institute for Health and Clinical Excellence (NICE) and the WHO have published guidelines emphasizing preoperative, intraoperative, and postoperative measures [18,19]. Table 1 summarizes key WHO guidelines for SSI prevention [19].

**Table 1:** Summary of the World Health Organization global guidelines for the prevention of surgical site infections This table displays important preoperative, intraoperative, and postoperative measures that should be considered for the prevention of surgical site infections during different surgical operations [19].

World Health Organization SSI Prevention Guidelines
Preoperative Measures:
Patients should bathe or shower
Shaving patients is not recommended
Use alcohol-based antiseptics (Chlorhexidine based) for skin preparation
Surgical hand preparation recommended: alcohol or antimicrobial soap-based wash
Use antibiotic prophylaxis when indicated (depends on the type of procedure)
Intraoperative Measures:
Maintain normal oxygen saturation in patients
Maintain normothermia
Blood glucose control recommended
Use sterile surgical equipment
Postoperative Measures:
Use of antibiotics prophylaxis post-surgery is not recommended
Use standard wound dressings

Standard systemic antibiotic prophylaxis regimen

The North American Spine Society (NASS) recommends intravenous (IV) perioperative antibiotics to prevent SSIs, yet it does not specify preferred antibiotics, dosages, or delivery routes [20]. First- or second-generation cephalosporins, particularly cefazolin, are commonly recommended due to their effectiveness against Gram-positive organisms [21]. Studies demonstrate that cefazolin significantly reduces infection rates compared to placebo and is effective in non-instrumented spine surgeries [22]. The rise of methicillin-resistant pathogens complicates prevention strategies. MRSA, first identified in 1961, presents challenges due to its resistance to multiple beta-lactam antibiotics [22]. Carriers of MRSA are more likely to develop SSIs, and there is a concerning increase in MRSA infections [23,24]. Thus, adjunctive prophylaxis is necessary to combat resistant bacterial strains.

Systemic versus topical use of vancomycin

The efficacy of IV vancomycin in reducing SSI rates remains unclear. In spinal surgery, a study suggests that topical vancomycin powder may be more effective than standard systemic antibiotics in reducing SSI rates [25]. The local application of antibiotics has historical precedence and offers advantages such as high drug concentrations at the surgical site with limited systemic absorption [26]. The use of topical vancomycin in preventing SSIs during spinal surgery shows mixed results. A meta-analysis indicates that topical vancomycin reduces infection rates caused by Gram-positive bacteria, including MRSA, but its effectiveness against Gram-negative infections remains uncertain [27]. Further research is needed to assess the potential risks of increased Gram-negative infections [28] and to establish the efficacy of topical vancomycin in clinical practice [29].

Economic burden of surgical site infections

SSIs impose significant financial burdens on healthcare systems. In the UK, an individual SSI can cost over £10,000, while deep SSIs may exceed £100,000 [30]. SSIs increase hospital stays, outpatient appointments, and reoperations, complicating patient recovery [30]. Implementing topical vancomycin in spinal surgery protocols may lead to substantial cost savings while reducing postoperative infection rates [11].

In conclusion, SSIs following spinal surgery represent a major challenge. As antibiotic resistance rises, exploring alternatives like topical vancomycin may be crucial in enhancing patient outcomes and reducing healthcare costs.

## Review

Methods 

Literature Search Strategy 

A thorough electronic literature search was conducted using three main databases to find potentially relevant articles. The databases systematically searched were Medline, Embase, and Web of Science. The literature search was performed using keywords or medical subject headings (MeSH) terms and included words similar to topical vancomycin, surgical site infections, spinal surgery, etc., as shown in Table 2. Comprehensive search terms were used to cover all phrases synonymous with topical vancomycin and SSIs. The search strategy used, specific to each database, is shown in Table 3.

**Table 2:** Key search terms

Keyword	Terms Used
Topical Vancomycin	“Vancomycin”, “local vancomycin”, “vancomycin powder”, “intrawound vancomycin”, “intraoperative vancomycin”, “Vancomycin Hydrochloride”
Surgical Site Infection	“Surgical wound infection”, “wound infection”, “postoperative wound infection”, “surgical wound”
Spine Surgery	“Spine”, “spinal column”, “spinal surgery”, “cervical vertebrae”, “thoracic vertebrae”, “lumbar vertebrae”, “spinal fusion”

**Table 3:** Search strategy

Database	Search Strategy
PubMed	Vancomycin/ or Topical Vancomycin.mp. or Surgical wound Infection/ Surgical site infection.mp. or Surgical Wound Infection/ Lumbar Vertebrae/ or Cervical Vertebrae/ or Spinal Fusion/ or spine surgery.mp. or Spine/ #1 AND #2 AND #3
Embase	wound infection/ or surgical infection/ or vancomycin/ or local vancomycin.mp. surgical site infection.mp. or surgical infection/ spinal surgery.mp. or spine surgery/ #1 AND #2 AND #3
Web of Science Core Collection	Vancomycin AND Surgical site infection AND Spine

Study Selection Criteria 

**Figure 1:**
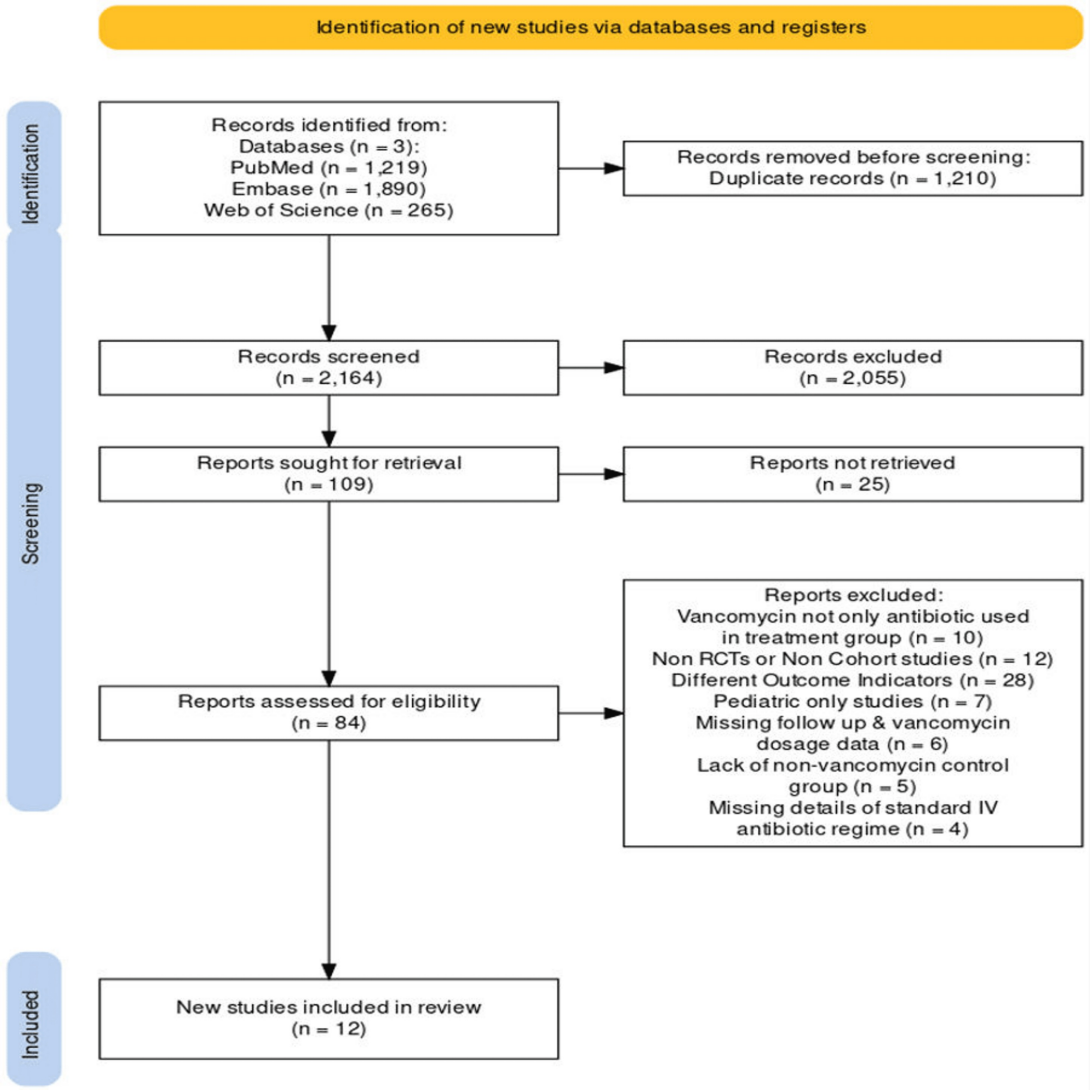
Flow chart displaying the process of screening and selection of included studies

Retrospective and prospective cohort studies and randomized controlled trials (RCTs) were selected based on their ability to answer the following research question: “Is topical vancomycin effective in the prevention of surgical site infections in spinal surgery patients?” The level of evidence for each study was determined using the 2011 Oxford level of evidence hierarchy [31]. The literature search was filtered on Medline, Embase, and Web of Science to find available RCTs or clinical trials, as these databases have this function. A PICO (population, intervention, control, and outcomes) framework was used to aid in defining and answering the intended research question. The population included any age patient who underwent spinal surgery with any type of spinal surgery included; the intervention is the application of topical vancomycin powder on the surgical site before wound closure, the control is standard perioperative system antibiotics, and the outcomes include postoperative surgical site infection rates and the infection-causing organism cultures or data results. Inclusion and exclusion criteria were defined to locate relevant studies that answer the research question and consider the interested outcome indicators, as shown in Table 4. The outcome indicators were the postoperative SSI rates in spinal surgery patients with or without the application of topical vancomycin and the microbiological findings associated with using vancomycin powder. The eligibility of articles involved screening the titles and abstracts, and any study potentially meeting inclusion criteria underwent a full-text review. After a full-text assessment, ineligible articles were excluded, and reference screening from each full-text article was done for completeness. Figure 1 summarizes the screening process in a flowchart diagram.

**Table 4:** Inclusion and exclusion criteria RCT: Randomised controlled trial

Inclusion Criteria	Exclusion Criteria
Studies published in any year	Unpublished studies
All RCTs, prospective and retrospective cohort studies on the topical use of vancomycin to prevent surgical site infections after spinal surgery.	Case-control studies, Case reports, systematic reviews, meta-analyses, and non-English studies.
Studies including human patients of any age and sex.	Animal studies and studies focusing only on one type of demographic such as paediatric patients
Studies involving patients with any type of spine condition or pathology (trauma, malignant, degenerative, infectious, or deformity) undergoing any type of spine surgery in any area of the spine (cervical, thoracic, lumbar).	Studies with patients who did not undergo spine surgery and/or have infections before procedure.
Studies with a treatment and control groups. Treatment groups receives intraoperative vancomycin powder before surgical wound closure and control groups did not receive vancomycin powder.	Studies lacking clearly defined treatment and control groups. Studies that use vancomycin powder after surgical wound closure.
Studies included patients that all received standard perioperative systemic antibiotic regimens.	Studies including patients that received other antibiotics that are not part of the standard antibiotic regimen, use of other topical antibiotics, and studies in which only one group received standard antibiotic regimen.
Studies that focus on outcomes including postoperative SSI incidence rates in both treatment and control groups, microbiological data, and safety and efficacy of topical vancomycin.	Studies that did not focus on the effect of topical vancomycin in SSI prevention, lack data on SSI rates, and involved pathogens not distinguished.
Studies reporting any side effects, adverse reaction, and dosages used of topical vancomycin.	Studies lacking information on topical vancomycin dosages used and/or missing data
Studies with patient follow up times of at least 1 month.	Studies with patient follow up time less than 1 month.

Data Extraction 

Relevant data from the included studies was extracted into an Excel spreadsheet (Redmond, USA) to summarize results and significant findings. The following data was collected: basic information on the studies (first authors, publication year, study type, level of evidence), patient characteristics (age and sex), sample sizes, surgery characteristics (type, location, indications), perioperative IV antibiotics used, topical vancomycin use (location, dosage, adverse reactions), SSI characteristics (type, incidence rates, pathogens involved), follow-up duration, and author’s conclusions. Statistical analysis software was not used in this review.

Quality Assessment

The quality of each study included in the critical review was assessed using the Critical Appraisal Skills Programme (CASP) checklists for RCTs and cohort studies [32,33]. These checklist tools were used to evaluate each included article and critically appraise the strengths and weaknesses of each study. Risk of bias assessment for the included RCTs was done using version 2 of the Cochrane risk-of-bias tool (RoB 2) [34]. Each domain in the tool is assigned a proposed risk of bias judgment, and the possible ratings include low risk of bias, some concerns, and high risk of bias [34]. An overall risk of biased judgment is assigned to the RCT based on each domain’s results. For the cohort studies included, bias assessment was done using the Risk of Bias in Non-randomized Studies - of Interventions (ROBINS-I) tool, which has very similar characteristics to the RoB 2 tool and rates studies as either low, moderate, and serious risk of bias, or critical risk of bias [35].

Results

Study Selection

The literature search identified 2,164 articles, which underwent title and abstract screening, as shown in Figure 1. After removing systematic reviews, meta-analyses, and studies that did not meet the inclusion criteria, 109 papers proceeded to full-text screening. Further exclusions were made due to the lack of SSI outcome data (25 studies), study types not being RCTs or cohort studies (12 studies), inclusion of additional antibiotics in the treatment group (10 studies), missing vancomycin dosage or follow-up data (six studies), studies including only pediatric patients (seven studies), inadequate control groups (five studies), and missing systemic antibiotic usage data (four studies). Following these exclusions, 12 studies [10,13,36-45] met the inclusion criteria and were included in the final review.

Study Characteristics

Of the 12 included studies, three were RCTs, two were prospective cohort studies, and seven were retrospective cohort studies. However, none of the RCTs were blinded. The included studies were published between 2011 and 2022, with most studies published between 2013 and 2017. The characteristics of each study, including the type of spinal surgery performed, the definition of SSI outcomes, and the level of evidence, are summarized in Table 5.

**Table 5:** The basic characteristics of the 12 included studies SSI: Surgical site infection.

Study (Author & Year)	Title	Study Type	Type of Surgery	Type of SSI involved	Outcome Definition	Level of Evidence
Madhuchandra et al. (2018) [40]	‘Efficacy of local vancomycin in preventing surgical site infections following spinal instrumentation’	Prospective cohort Study	Spinal instrumentation surgery	Superficial and Deep	SSI rate and microbiological data	2
Hey et al. (2017) [38]	‘Is intraoperative local vancomycin powder the answer to surgical site infections in spine surgery?’	Retrospective cohort comparative study	Elective spine surgery	Superficial and Deep	Rate of SSI and microbiological data	3
Heller et al. (2015) [39]	‘Intrawound vancomycin powder decreases staphylococcal surgical site infections following posterior instrumented spinal arthrodesis’	Retrospective historical cohort study	Posterior instrumented spinal arthrodesis surgery	Superficial and Deep	Rate of SSI, Risk factors, microbiological data	3
Caroom et al. (2013) [37]	‘Intrawound vancomycin powder reduces surgical site infections in posterior cervical fusion’	Retrospective comparative cohort study using prospectively collected data	Posterior cervical decompression and instrumentation surgery	Superficial and Deep	Rate of SSI and causative organism cultures	3
Sweet et al. (2011) [36]	‘Intrawound Application of Vancomycin for prophylaxis in instrumented thoracolumbar fusions’	Retrospective cohort study from a single institution	Thoracic and lumbar posterior instrumented spinal fusions	Deep	Rate of SSI, wound complications, vancomycin drug levels, microbiological data	3
Ushirozako et al. (2021) [41]	‘Impact of intrawound vancomycin powder on prevention of surgical site infection after posterior spinal surgery’	Retrospective cohort study	Posterior spinal surgery	Posterior spinal surgery	Posterior spinal surgery	3
Horii et al. (2018) [13]	‘Does intrawound vancomycin powder reduce surgical site infection after posterior instrumented spinal surgery? A propensity score-matched analysis’	Multicentre retrospective cohort study	Posterior instrumented spinal surgery	Not classified	SSI rate and microbiological cultures	3
Gaviola et al. (2016) [42]	‘A retrospective study on the protective effects of topical vancomycin in patients undergoing multilevel spinal fusion’	Retrospective cohort study	Multilevel spinal fusion surgery	Superficial and Deep	Rate of SSI, Risk factors, and causative organism cultures	3
Prasad et al. (2022) [45]	‘Study on effect of local application of vancomycin powder on surgical site infection rate in patients undergoing instrumented spinal fusion surgeries’	Prospective cohort Study	Instrumented spinal fusion surgery	Superficial and Deep	SSI rate	2
Salimi et al. (2022) [44]	‘Local vancomycin therapy to reduce surgical site infection in adult spine surgery: a randomized prospective study’	Prospective randomized controlled trial	Open spinal surgery in the cervical, thoracic, and lumbosacral regions. Instrumented and non-instrumented cases.	Superficial and Deep	SSI rate and causative organism cultures	2
Mirzashahi et al. (2017) [10]	‘Intrawound application of vancomycin changes the responsible germ in elective spine surgery without significant effect on the rate of infection: a randomized prospective study’	Prospective randomized controlled trial	Elective spine surgery	Superficial or Deep	SSI rate and microbiological data	2
Tubaki et al. (2013) [43]	‘Effects of using intravenous antibiotic only versus local intrawound vancomycin antibiotic powder application in addition to intravenous antibiotics on postoperative infection in spine surgery in 907 patients’	Prospective randomized controlled trial	Open spine surgery at any level. Includes instrumented and non-instrumented cases.	Superficial and Deep	Rate of SSI and causative organism cultures	2

Patient Demographics

A total of 9,224 patients were included across the 12 studies, with 3,756 patients receiving intraoperative topical vancomycin powder and 5,468 patients comprising the control group. The sample sizes ranged from 80 to 2,859 patients per study. The characteristics of the patients, including age, sex, body mass index (BMI), and comorbidities, are presented in Table 6. All patients in both groups received standard systemic antibiotic prophylaxis before surgery, with perioperative treatment details summarized in Table 7.

**Table 6:** Patient characteristics of the 12 included studies Yrs.: Years, TG: Treatment group, CG: Control group, DM: Diabetes mellitus, TB: Tuberculosis, HB: Hepatitis B, RA: Rheumatoid arthritis, IHD: Ischemic heart disease, COPD: Chronic obstructive pulmonary disease, UTI: Urinary tract infection, CSF: cerebrospinal fluid, Kg: kilogram, and m2: metre squared.

Study (Author & Year)	Mean age (yrs.)		Male sex (%)		Mean BMI (Kg/m^2^)		Revision Surgery (%)		Reported risk factors and/or comorbidities
	TG	CG	TG	CG	TG	CG	TG	CG	
Madhuchandra et al. (2018) [40]	50.0	52.0	51.0	47.0	Not reported		Not reported		Not reported
Hey et al. (2017) [38]	45.0	48.0	44.0	53.0	23.9	24.4	2.0	4.0	DM, hypertension, IHD, hyperlipidaemia, smoking, alcohol intake,
Heller et al. (2015) [39]	55.3	49.1	45.3	49.3	31.6	30.7	50.0	39.8	DM, cardiovascular disease, hypertension, respiratory disease, smoking, blood transfusion
Caroom et al. (2013) [37]	59.8	56.4	Not reported		>30 in 50% of cohort	>30 in 42% of cohort	Not reported		DM, obesity
Sweet et al. (2011) [36]	56.0	53.0	49	52	Not reported		23	13	DM, smoking, blood transfusion
Ushirozako et al. (2021) [41]	63.3	61.4	37.7	42.5	23.7	23.1	15.2	8.0	DM, hypertension, COPD, asthma, chronic renal dysfunction, collagen disease
Horii et al. (2018) [13]	68.5	65.0	47.0	46.3	23.2	23.5	39.0	16.0	DM, smoking, steroid use, haemodialysis, immunosuppressant use
Gaviola et al. (2016) [42]	62.0	55.0	56.0	56.7	26.7	28.2	31.0	39.5	DM, obesity, renal dysfunction, tobacco use
Prasad et al. (2022) [45]	38.3	45.2	20.0	23.3	Not reported		0	0	Tobacco use, Alcoholism, Hypertension, DM, TB, HB, RA
Salimi et al. (2022) [44]	51.7	52.4	42.2	44.7	>30 in 12.3% of cohort	>30 in 13.3% of cohort	Not reported		DM, hypertension, IHD, COPD, UTI, smoking, neurological defect, CSF leak, blood transfusion
Mirzashahi et al. (2017) [10]	Not reported		35.5	35.5	>30 in 1.03% of cohort	>30 in 1.60% of cohort	Not reported		DM, smoking, obesity, use of instrumentation
Tubaki et al. (2013) [43]	44.5	46.6	49.0	50.7	Not reported		Not reported		DM, hypertension, IHD, asthma

**Table 7:** Treatment characteristics of the 12 included studies IV: Intravenous, g: grams, mg: milligrams, kg: kilograms.

Study (Author & Year)	Preoperative Prophylaxis (control)	Intraoperative Prophylaxis (intervention)	Postoperative Prophylaxis	Site of Vancomycin delivery	Post-operation Follow-up time (months)	Reported Adverse events
Madhuchandra et al. (2018) [40]	IV ceftriaxone and sulbactum combination. Dosage not recorded.	1g vancomycin powder before wound closure	Standard IV antibiotics for 2-3 days then oral antibiotics for another 5 days	Below fascia layer	3	None
Hey et al. (2017) [38]	1 g IV cefazolin. If penicillin allergy given 1 g IV vancomycin.	1 g vancomycin powder before wound closure	IV cefazolin for 2 days	Subfascial space	12	None
Heller et al. (2015) [39]	20 mg/kg IV cefazolin within 1 hour of incision. Repeat dosing every four hours during procedure.	0.5 g – 2 g vancomycin powder before wound closure	1 g IV cefazolin every 8 hours for 1 day	Fascia, muscle, subcutaneous tissue	3	Pseudoarthrosis
Caroom et al. (2013) [37]	IV antibiotics according to institutional policies	1 g vancomycin powder before wound closure	IV antibiotics for 1 to 2 days. According to policies.	Subfascial layer, along bone graft & instrumentation	6	None
Sweet et al. (2011) [36]	2 g IV cefazolin within 1 hour of incision	2 g vancomycin powder before wound closure	IV cefazolin for 1 day	Directly on surgical wound	30	Pseudoarthrosis
Ushirozako et al. (2021) [41]	1^st^ generation cephalosporin. If allergic given penicillin. Dosage not recorded. Repeat dosing for surgeries longer than 3 hours or after significant blood loss.	1g vancomycin powder before wound closure	1^st^ generation cephalosporins for 2 days	Implants, muscles, and autografts	12	None
Horii et al. (2018) [13]	IV cefazolin before incision. If allergic, given clindamycin. Dosage not recorded.	1 or 2 g vancomycin powder before wound closure	IV cefazolin for at least l day	Implants, muscles, fascia, subcutaneous tissue	12	None
Gaviola et al. (2016) [42]	2 g IV cefazolin given 1 hour of incision. Repeat dosing every 3 hours during surgery.	2 g vancomycin powder before wound closure	Not reported	Soft tissue, implants, bones	3	None
Prasad et al. (2022) [45]	1 g IV ceftriaxone within 1 hour of surgical incision	500 mg – 1g vancomycin powder before wound closure	1 g IV ceftriaxone every 8 hours for 1 day	50% dose above the deep lumbar fascia and 50% dose below fascia	15	Severe lower back ache and pain
Salimi et al. (2022) [44]	2 g of IV cephalosporin within 2 hours before surgery, another 1 g given if surgery longer than 2 hours	1g – 2g vancomycin powder before wound closure	1g IV cephalosporin every 8 hours for 1 day	Muscles, bones, subcutaneous tissue but not skin, dura, or fusion site	3	None
Mirzashahi et al. (2017) [10]	1 or 2 g IV cephazolin 20 minutes before incision. If allergic give clindamycin. Repeat dosing every 3 hours during surgery or after significant blood loss.	1 or 2 g vancomycin powder before wound closure	Not reported	Over the fascia	15	None
Tubaki et al. (2013) [43]	750 mg IV cefuroxime just before incision	1 g vancomycin powder before wound closure	750 mg IV cefuroxime every 8 hours for 1 day	Fascia, muscle, subcutaneous tissue	12	None

Surgical Site Infection (SSI) Incidence

Across the included studies, a total of 279 patients developed SSIs, with 85 cases occurring in the vancomycin treatment group and 194 in the control group. The overall infection rate was determined to be 2.10%. The statistical significance of the impact of topical vancomycin on surgical site infection (SSI) prevention was determined using a p-value threshold of <0.05. Five studies [36-40] demonstrated a statistically significant reduction in SSI rates following the application of vancomycin powder during spinal surgery compared to the control group. Three studies [13,41,42] reported a reduction in SSI incidence in the vancomycin group that did not reach statistical significance. The remaining four studies [10,43-45], including all RCTs, found no statistically significant difference in SSI rates between the treatment and control groups. A summary of sample sizes, SSI incidence rates, and study conclusions is provided in Table 8.

**Table 8:** The results and conclusions of the 12 included studies SSI: Surgical site infection, N: number of patients.

Study (Author & Year)	Total Patient Cohort (n)	Vancomycin Group (n)	Control Group (n)	SSIs in vancomycin group (%)	SSIs in control group (%)	Authors’ Conclusions
Madhuchandra et al. (2018) [40]	80	40	40	2.5	12.5	Statistically significant reduction in infection rate between treatment and control group. Topical vancomycin is effective in reducing rates of infection after spine surgery.
Hey et al. (2017) [38]	389	117	272	0.9	6.3	Statistically significant decrease in SSI rates in treatment group compared to control. Topical vancomycin reduces risk and morbidity of SSI in spine surgery.
Heller et al. (2015) [39]	683	342	341	2.6	8.9	Statistically significant reduction in SSI rates in treatment group compared to control. Local vancomycin is active against SSIs.
Caroom et al. (2013) [37]	112	40	72	0	15	Statistically significant reduction in SSI rates in treatment group compared to control. Local vancomycin powder can reduce incidence of postoperative SSIs.
Sweet et al. (2011) [36]	1732	911	821	0.2	2.6	Statistically significant reduction in SSI rates in treatment group compared to control. Topical vancomycin reduces infection risk in spine surgery patients.
Ushirozako et al. (2021) [41]	1261	623	638	3.3	5.2	Decreased incidence of SSIs in treatment group compared to control but results not statistically significant.
Ushirozako et al. (2021) after Propensity Score-Matched Analysis [41]	888	444	444	2.7	5.4	Statistically significant reduction of postoperative SSI rate in treatment group compared to control group. Topical vancomycin was useful in reducing risk of SSI by half after spine surgery.
Horii et al. (2018) [13]	2859	694	2165	1.73	0.97	Decreased SSI rates in treatment group compared to control group, but results not statistically significant. Rate of infections caused by Staphylococcal species was lower in the treatment group.
Horii et al. (2018) after Propensity Score-Matched Analysis [13]	1014	507	507	1.58	1.78	Decreased SSI rate in treatment group compared to control group but difference is not statistically significant.
Gaviola et al. (2016) [42]	326	116	210	5.2	11.0	Decreased SSI incidence in treatment group compared to control but results not statically significant. Topical vancomycin and IV cefazolin are protective against SSI development.
Prasad et al. (2022) [45]	120	60	60	3.3	1.7	Topical vancomycin did not reduce the incidence of SSIs in spine surgery. Incidence of SSIs in the treatment group could be an incidental finding due to the low sample size.
Salimi et al. (2022) [44]	375	187	188	13.1	13.6	No statistically significant difference between treatment and control group in SSI rates. Topical vancomycin has no effect on SSI.
Mirzashahi et al. (2017) [10]	380	193	187	5.2	2.7	Statistically no significant difference in SSI rates between both groups. Treatment changed causative pathogen.
Tubaki et al. (2013) [43]	907	433	474	1.61	1.68	Statistically no significant difference in SSI rates between both groups. Topical vancomycin did not significantly prevent SSI incidence.

Microbiological Findings

**Figure 2:**
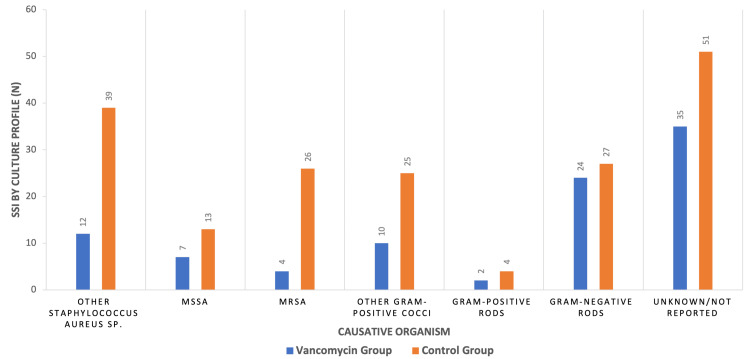
Microbiological results of the 12 included studies. A total of 279 Surgical site infections (SSIs) are classified by the causative organisms in both the vancomycin treatment and control groups N: number of SSIs, MSSA: Methicillin-sensitive *Staphylococcus aureus*, MRSA: Methicillin-resistant *Staphylococcus aureus*, SSI: Surgical site infection

The microbiological analysis of SSIs reported in the included studies is illustrated in Figure 2. The most frequently identified pathogens included *Staphylococcus aureus*, coagulase-negative *staphylococci*, and gram-negative bacteria.

Risk of Bias Assessment

**Figure 3:**
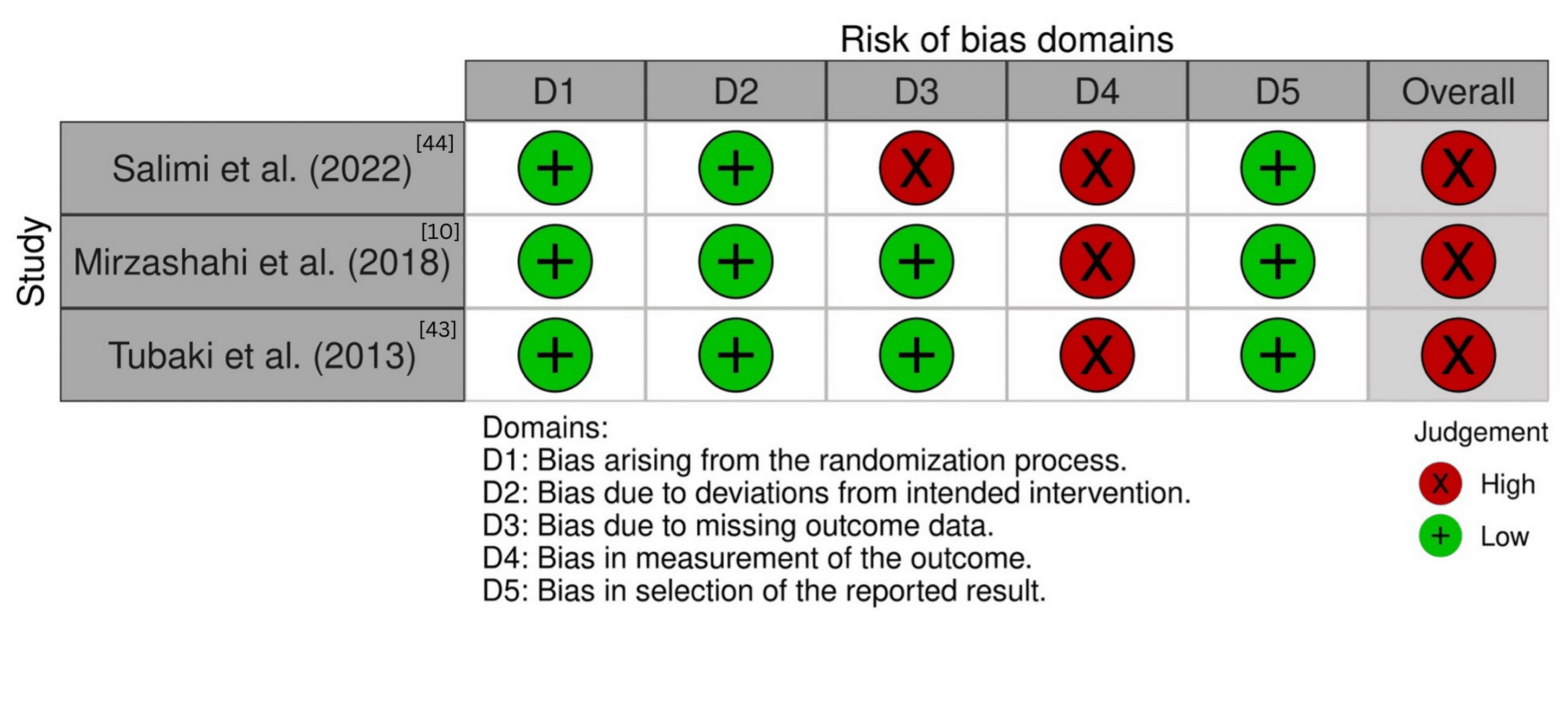
Risk of bias assessment for included randomised controlled trials using RoB 2 tool

**Figure 4:**
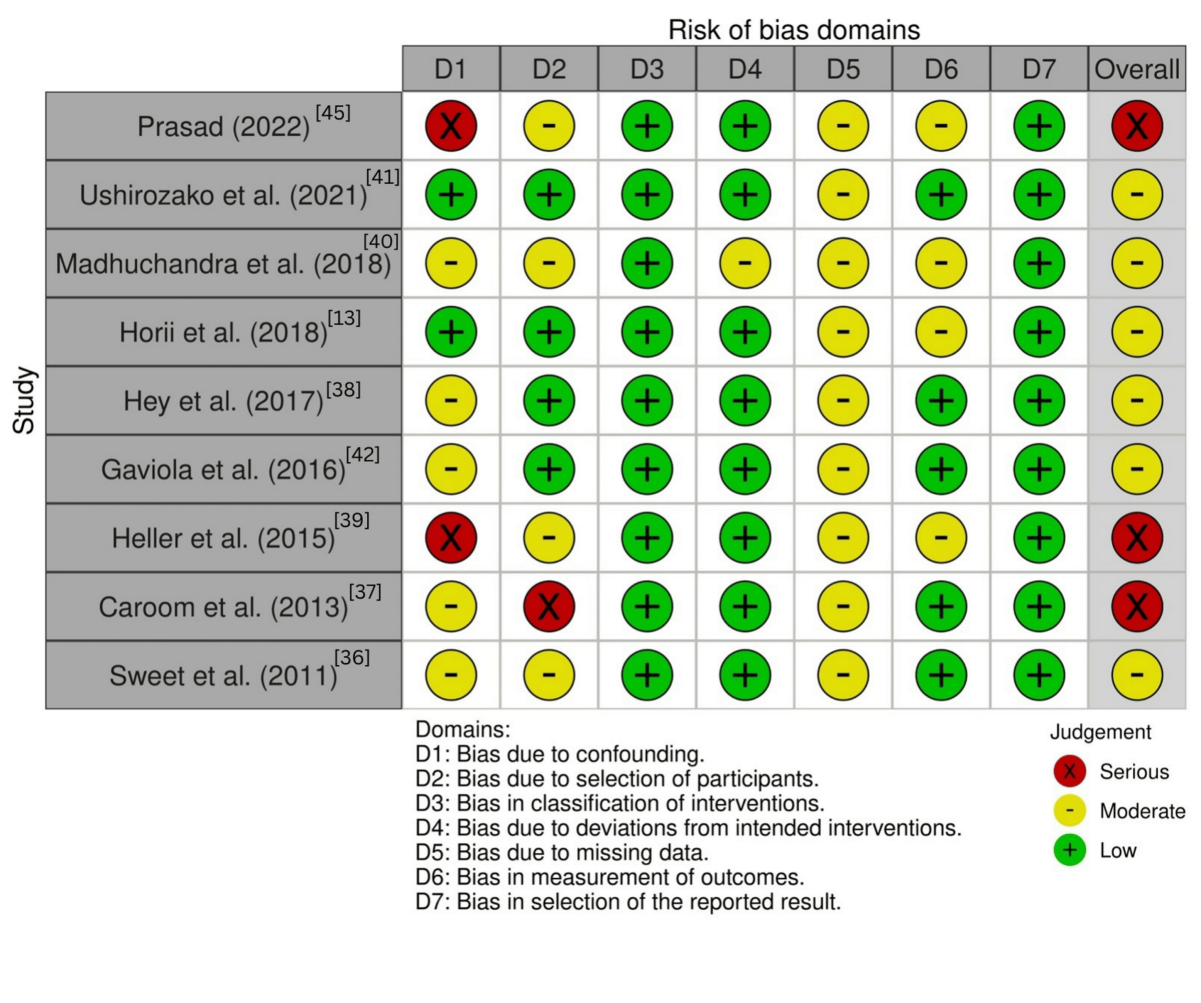
Risk of bias assessment for included cohort studies using ROBINS-I tool

The quality assessment of the included studies was conducted using standardized tools. The risk of bias for the RCTs was evaluated using the RoB 2 tool [34], and the results are shown in Figure 3. Cohort studies were assessed using the ROBINS-I tool [35], with the findings presented in Figure 4. The CASP checklists for RCTs and cohort studies were used to further evaluate study quality, generating the results shown in Tables 9, 10 [32,33]. 

**Table 9:** Quality assessment of the included randomised control trials using the CASP checklist Questions part of the checklist: 1. Did the study address a clearly focused research question? 2. Was the assignment of participants to interventions randomised? 3. Were all participants who entered the study accounted for in the conclusion? 4. Were the participants ‘blind’ to the intervention they were given? Were the investigators ‘blind’ to the intervention they were giving to participants? Were the people assessing/analysing outcome/s ‘blinded’? 5. Were the study groups similar at the start of the randomised control trial? 6. Apart from the experimental intervention, did each study group receive the same level of care (that is, were they treated equally)? 7. Were the effects of the intervention reported comprehensively? 8. Was the precision of the estimate of the intervention or treatment effect reported? 9. Do the benefits of the experimental intervention outweigh the harms and costs? 10. Can the results be applied to your local population/in your context? 11. Would the experimental intervention provide greater value to the people in your care than any of the existing interventions?

Study	1	2	3	4	5	6	7	8	9	10	11
Salimi et al. (2022) [44]	Yes	Yes	No	No	Yes	Yes	No	No	Can’t tell	Yes	Can’t tell
Mirzashahi et al. (2017) [10]	Yes	Yes	Yes	No	Yes	Yes	No	No	Can’t tell	Yes	Can’t tell
Tubaki et al. (2013) [43]	Yes	Yes	Yes	No	Yes	Yes	No	No	Can’t tell	Yes	Can’t tell

**Table 10:** Quality assessment of the included cohort studies using the CASP checklist CASP: Critical Appraisal Skills Program Questions part of the checklist: 1. Did the study address a clearly focused issue? 2. Was the cohort recruited in an acceptable way? 3. Was the exposure accurately measured to minimise bias? 4. Was the outcome accurately measured to minimise bias? 5. (a) Have the authors identified all important confounding factors? (b) Have they taken account of the confounding factors in the design and/or analysis? 6. (a) Was the follow up of subjects complete enough? (b) Was the follow up of subjects long enough? 9. Do you believe the results? 10. Can the results be applied to the local population? Questions 7 and 8 were excluded as they were answered in other sections of the results.

Study	1	2	3	4	5 (a)	5 (b)	6 (a)	6 (b)	9	10	11	12
Prasad et al. (2022) [45]	Yes	Yes	Yes	No	No	No	Yes	Yes	No	Can’t tell	Yes	Can’t tell
Ushirozako et al. (2021) [41]	Yes	Yes	Yes	Yes	Yes	Yes	Yes	Yes	Yes	Can’t tell	Yes	Yes
Madhuchandra et al. (2018) [40]	Yes	Yes	Yes	No	No	Yes	Yes	Yes	Yes	Can’t tell	Yes	No
Horii et al. (2018) [13]	Yes	Yes	Yes	Yes	Yes	Yes	Yes	Yes	Yes	Can’t tell	Yes	Can’t tell
Hey et al. (2017) [39]	Yes	Yes	Yes	Yes	Yes	No	Yes	Yes	Yes	Can’t tell	Yes	No
Gaviola et al. (2016) [42]	Yes	Yes	Yes	Yes	Yes	Yes	Yes	Yes	Yes	Can’t tell	Yes	Can’t tell
Heller et al. (2015) [39]	Yes	Yes	Yes	No	Yes	No	Yes	Yes	Yes	Can’t tell	Yes	Can’t tell
Caroom et al. (2013) [37]	Yes	No	Yes	Yes	No	Yes	Yes	Yes	Yes	No	Yes	Can’t tell
Sweet et al. (2011) [36]	Yes	Yes	Yes	Yes	No	No	Yes	Yes	Yes	Can’t tell	Yes	Yes

Discussion

This critical review evaluates the effectiveness of topical vancomycin in preventing surgical site infections (SSIs) in spinal surgery patients. The intraoperative use of vancomycin powder remains controversial due to mixed evidence in current literature. This review aims to clarify the ambiguity surrounding vancomycin use in spinal surgery by interpreting study results, assessing their validity, evaluating biases, and examining strengths and weaknesses.

Types of Studies

Among the included studies, three are randomized controlled trials (RCTs), two are prospective cohort studies, and seven are retrospective cohort studies. Observational studies were included to provide a comprehensive approach to this review due to the lack of clinical trials or RCTs explicitly addressing the research question. The level of evidence in these studies was either I or II, with RCTs and prospective cohort studies providing higher levels of evidence than retrospective cohort studies. However, the included RCTs were not double-blinded. The absence of blinding increases the risk of performance bias, where knowledge of treatment allocation may influence intraoperative or postoperative care. Additionally, detection bias may occur if outcome assessors are aware of group assignments, potentially affecting the objectivity of infection diagnosis. These factors may overestimate or underestimate the true effect of topical vancomycin on SSI rates. The RCTs also lacked registration numbers and were conducted at single centers. All observational studies were also performed at single treatment centers except for Horii et al. [13], which was a multicenter study incorporating spinal surgery patients from multiple hospitals.

Different Types of Spine Surgery Involved

The studies included in this review examined the use of topical vancomycin in various types of spinal surgery, including decompression procedures, fusion surgeries, and complex spinal reconstructions. Spinal fusion surgery is particularly associated with a high risk of SSIs due to prolonged operative time and extensive tissue dissection [45]. Several studies [36,42,45] suggest that vancomycin application may be more beneficial in high-risk procedures, while its efficacy in lower-risk surgeries remains uncertain.

Variability in the Definition of Surgical Site Infection

Only four studies [13,38,41,42] diagnosed patients with SSIs based on the definition established by the CDC and classified them into their respective categories. The definition outlined by the CDC states that SSIs occur within 30 days (or 90 days if an implant is used) from an operation and involve the skin/subcutaneous tissue of the incision [2]. The CDC’s definition of SSI provides an established and recognizable approach to diagnosing SSIs during surgical operations. Two studies [39,40] used unique measures to define SSIs, and the six other studies [10,36,37,43-45] were missing information on how SSIs were defined and diagnosed in their study protocols. Thus, there is a lack of a generalisable and comprehensive standard to which the effectiveness of topical vancomycin can be examined, as subtle differences in infection definition can create diverse responses in treatment outcomes. Also, it makes it more probable for researchers to be biased when deciding if a patient has an SSI or not, causing expectancy bias. 

Baseline Patient Characteristics for Each Study

Most of the included studies did not effectively control for potential risk factors or other patient characteristics between treatment cohorts, which can limit the generalisation of the results. However, seven studies [10,38,41-45] also had predefined exclusion criteria, such as patients with current infections, use of immunosuppressants, radiation or chemotherapy treatments, current use of antibiotics, allergies to vancomycin, and inability to follow-up, as a method to limit risk factors. However, these factors still need to be controlled to prevent potential skewing of results and to demonstrate a clear correlation between topical vancomycin use and prevention of SSIs in spinal surgery. Future studies should prioritize investigating risk factors to establish a better patient selection criterion and reduce confounding.

Dosage, Delivery, and Safety Profile

The studies sustained consistency in the treatment cohorts by having all patients receive topical vancomycin powder before the closure of the surgical wound. However, the dose received by spinal surgery patients ranged from 500mg to 2g, with most of the included studies applying a dose of either 1g or 2g. Currently, there is no set standard of how much vancomycin should be applied, and information on the safety of delivery based on optimal dosing needs to be included. Variations in the dosage and delivery site of topical vancomycin may contribute to the heterogeneity of outcomes across studies. Lower doses might result in subtherapeutic concentrations at the surgical site, reducing efficacy in preventing SSIs. Conversely, higher doses may enhance antimicrobial coverage but could also increase the risk of local toxicity, such as impaired wound healing or seroma formation. Commonly, the amount of applied vancomycin powder is left in the hands of the surgeons themselves and their clinical expertise, adding to procedural bias. Further pharmacological studies are needed to determine the optimal dose of topical vancomycin to prevent SSIs in spinal surgery effectively. Like dosage, there was variability in the site of delivery of intrawound vancomycin powder in the included studies. However, common delivery sites included muscle, fascia, subcutaneous tissue, bones, and implants. Sweet et al. [36] were unique in reporting the application of vancomycin powder directly onto the surgical wound. As no other study adopts this approach, the validity of this method should be considered with precaution. There is no agreement as to exactly where vancomycin powder should be applied for optimal SSI prevention. From the included studies, very few adverse events were reported with the use of topical vancomycin in spinal surgery.

Effects of Topical Vancomycin on Postoperative Surgical Site Infection Rates

Topical vancomycin has been investigated for its potential to reduce postoperative SSIs in spinal surgery, but the evidence remains mixed. Four studies [10,43-45] found no significant effect on SSI rates, with some reporting increased SSI rates in the vancomycin group. Among these, three were RCTs [10,43,44], which had strengths in their randomisation processes and outcome reporting. However, all three studies suffered from performance bias due to unblinded patients and outcome evaluators. The Salimi et al. [44] study experienced additional attrition bias, as patients were lost due to incomplete data or missing consent. The prospective cohort study [45] had a very small sample size, limiting the statistical power and generalisability of its findings.

In contrast, three retrospective cohort studies [13,41,42] found a reduction in SSI rates in the vancomycin group, although the results were not statistically significant. These studies had the largest sample sizes, with a combined total of 4,446 patients. Two studies [13,41] used propensity score matching to adjust for confounding factors, enhancing the reliability of their findings. Ushirozako et al. [41] found a statistically significant reduction in SSI rates when analyzing the matched groups, suggesting that vancomycin may be beneficial in certain contexts.

Five studies [36-40] reported significant reductions in SSI rates in the vancomycin group. Sweet et al. [36] was one of the first to demonstrate a clear benefit of vancomycin in preventing SSIs. However, studies like Caroom et al. [37] had small sample sizes, and some studies experienced attrition bias, which may have influenced results. Despite the general support for vancomycin’s effectiveness, the variability in study designs, sample sizes, and biases highlights the need for larger, well-controlled trials to conclusively determine its role in reducing SSIs in spinal surgery.

Antimicrobial Resistance Considerations 

Topical vancomycin is effective against Gram-positive organisms, including MRSA and MSSA [24], but ineffective against Gram-negative pathogens [24]. Microbiological results show fewer infections caused by Gram-positive organisms in the vancomycin group, with a slight reduction in Gram-negative SSIs, though insignificant. Some studies suggest vancomycin may alter the microbial profile [10,13,38,41,43,44], raising concerns about antibiotic resistance. While no study in this review provided direct evidence of resistance emergence, theoretical concerns persist regarding selective pressure leading to increased resistance among Gram-positive pathogens. This not only undermines the efficacy of vancomycin for systemic infections but also poses a broader public health risk by contributing to the global rise in antimicrobial resistance. Given the increasing burden of resistant infections on healthcare systems, careful consideration and further surveillance are needed before adopting widespread prophylactic use of topical vancomycin.

Strengths, Limitations, and Clinical Implications of Topical Vancomycin in Spinal Surgery

This review highlights the potential of topical vancomycin in preventing surgical site infections (SSIs) in spinal surgery, emphasizing its benefits against resistant bacterial strains and low complication rates. The review utilized a comprehensive search strategy and rigorous data extraction. However, the included studies had significant limitations, including retrospective designs, lack of randomization, small sample sizes, and varying patient characteristics and surgical protocols, all of which introduce bias and confounders. The absence of double-blinding in some RCTs further reduces the reliability of the results. The review recommends more large-scale studies with controlled cohorts to confirm vancomycin's effectiveness. Clinicians should carefully weigh the pros and cons of using vancomycin on a case-by-case basis, and future research should explore optimal dosing, delivery methods, and its efficacy against a broader range of pathogens. While promising, further evidence is needed before widespread adoption in spinal surgery or other surgical fields.

*Strengths and Limitations of This Review*

This review is comprehensive, utilizing multiple databases, a well-organized search strategy, and predefined inclusion/exclusion criteria. A wide range of keywords was employed, ensuring extensive coverage of studies. Data extraction was thorough, and appropriate quality assessment measures were applied. Results were clearly presented.

However, the review has limitations. Most of the included studies are retrospective, with inherent biases such as lack of randomization and poor control of confounding factors. The included RCTs suffer from biases and weak study designs, and there is considerable variability across studies in patient characteristics, surgical types, SSI definitions, antibiotic protocols, and follow-up durations. These differences can affect the assessment of vancomycin’s effectiveness in preventing SSIs. Additionally, many studies had small sample sizes and did not calculate power. A meta-analysis could have provided more robust evidence, but the decision not to conduct one was due to the heterogeneity of the studies included. Moving forward, a meta-analysis could be considered. The review's methodology could be strengthened by incorporating a secondary reviewer and expanding the literature search to include more diverse studies, including those that are translated or previously excluded.

Future Studies

Large-scale, randomized, double-blinded studies are necessary to validate the efficacy of vancomycin powder in spinal surgery. These studies should be sufficiently powered, use similar patient groups, control or adjust for confounding variables, and standardize SSI definitions. Future research could clarify the risk of Gram-negative infections and antimicrobial resistance. Investigating the pharmacokinetics of topical vancomycin and potential complications will be essential. Overall, while the review does not conclude that vancomycin is effective, further studies may confirm its value.

## Conclusions

Based on the evidence of this review, it cannot be concluded that there are benefits to the use of topical vancomycin in preventing surgical site infections (SSIs) during spinal surgery; therefore, further high-quality clinical trials and RCTs are needed to confirm the effectiveness of this intervention. The quality of each study was diverse, with strengths and weaknesses. Risk of bias was a common theme, with most studies lacking control over confounding factors, selection bias, performance bias, and bias due to missing data. Differences in sample sizes, spinal procedures, patient risk factors, comorbidities, perioperative antibiotics, follow-up times, vancomycin dosages, and delivery sites limit the potential to show a clear relationship between topical vancomycin and the prevention of SSIs. Unclear definitions of SSIs and the lack of information on microbial changes caused by vancomycin powder decrease the validity of these studies.
